# Evidence-based guidelines for the management of diabetic foot problems

**DOI:** 10.1186/1757-1146-4-S1-I11

**Published:** 2011-05-20

**Authors:** Jonathan Shaw

**Affiliations:** 1 Baker IDI Heart and Diabetes Institute Level 4, 99 Commercial Road, Melbourne VIC 3004

## 

Management of diabetic foot problems presents a series of complex challenges for patients and for health care professionals. Appropriate guidance on best practice is essential to delivering high-quality care. The NHMRC guidelines for the management of diabetic foot problems in primary care have just been updated to address this issue. The guidelines have been developed using systematic reviews of the literature, and broad consultation of organisations representing the relevant health care professionals and patients. The development of the guidelines, the evidence considered and the recommendations developed will be reviewed.

